# Emergency Department Nurses’ Experiences of a Mindfulness Training Intervention: A Phenomenological Exploration

**DOI:** 10.1177/08980101221100091

**Published:** 2022-05-16

**Authors:** Sofia Trygg Lycke, Fanny Airosa, Lena Lundh

**Affiliations:** Specialist nurse and health care development leader, Academic Primary Health Care Centre, Region Stockholm, Sweden; Division of Nursing, Department of Neurobiology, Care Sciences and Society, 27106 Karolinska Institutet, Stockholm, Sweden; Quality developer, 83223 Ersta Hospital, Stockholm, Sweden; Specialist nurse and head of the Lifestyle Unit, Academic Primary Health Care Centre, Region Stockholm, Sweden; Researcher, Division of Family Medicine and Primary Care, Department of Neurobiology, Care Sciences and Society, 27106 Karolinska Institutet, Stockholm, Sweden

**Keywords:** holistic nursing, compassion, mindfulness, loving-kindness, qualitative interviews

## Abstract

**Purpose:** The purpose of this study was to explore emergency care nurses’ experiences of an intervention to increase compassion and empathy and reduce stress through individual mindfulness training delivered via workshops and a smartphone application. We also explored how the nurses felt about the practical and technical aspects of the intervention. **Design:** Qualitative interview study. **Method:** Individual interviews were conducted with eight of the 56 participants in the intervention study and used phenomenological analysis to illuminate how they made sense of their lived experiences of mindfulness training. **Findings:** Three themes illuminated the nurses’ experiences: becoming aware, changing through mindfulness, and gaining the tools for mindfulness through workshops and the mobile application. The first two themes expressed personal experiences, whereas the third expressed experiences of the practical and technical aspects of the intervention. Most nurses found the mobile application easy to use and effective. **Conclusions:** Emergency care nurses can feel that the awareness and changes that come with mindfulness training benefit them, their colleagues, and the patients for whom they care. The findings also provide insights into the challenges of practicing mindfulness in a busy emergency care setting and into the practical aspects of using a smartphone application to train mindfulness.

## Background

Nursing has historically valued holistic approaches to health care (Blaszko Helming et al., 2020). In the philosophy and practice of holistic nursing, one core value includes self-care, self-reflection, and self-development (AHNA, 2021; Blaszko Helming et al., 2020). Nurses should take responsibility for cultivating this value by devoting time and attention to their well-being (Blaszko Helming et al., 2020). This is important for their mental and physical resilience and for maintaining a healthy working environment (Blaszko Helming et al., 2020). It is also crucial to nurses’ ability to see patients as whole people and provide holistic, compassionate care (AHNA, 2021; Blaszko Helming et al., 2020).

### Stress in the Emergency Hospital Care Setting

In an emergency care setting, nurses can find it particularly challenging to maintain self-compassion and self-care (Hunsaker et al., 2015). Emergency care nurses have multiple sources of stress, including short-term patient admissions, the need to make quick decisions, the introduction and application of new technology, staffing shortages, long working hours, and limited resources (Aiken et al., 2002; Jenkins & Warren, 2012). Stress can make it hard for them to focus on patient needs, which in turn can negatively affect the quality of care and their own work satisfaction (Kelly et al., 2015; Salvarani et al., 2019). Previous studies indicate that nurses working in an emergency context have a high risk of stress-related illness and burnout (Kelly et al., 2015; Salvarani et al., 2019).

### Reactions to Encountering the Suffering of Others

Regular encounters with others who are suffering can also be a source of stress for emergency care nurses. As part of their work, nursing staff meet suffering patients on a daily basis. According to Singer and Klimecki (2014), people encountering the suffering of others can react in different ways. The more constructive way is with empathy and compassion. Those who show compassion are not distant or overwhelmed by the suffering of others, but they also do not overidentify with the situation or the feelings that the situation arouses (Seppala et al., 2014). Empathetic and compassionate encounters can improve the compassionate person's well-being and promote his or her resilience (Singer & Klimecki, 2014). Reacting with compassion may lead nursing staff to feel calmer and experience less stress and can increase the quality, efficiency, and safety of care (Halpern, 2014).

On the other hand, people may react to the suffering of others with empathic distress, a state characterized by adopting an egocentric attitude and experiencing negative feelings such as stress that may lead to poor health, burnout, and distancing, non-social behavior (Singer & Klimecki, 2014). Nursing staff who feel empathic distress may exhibit a non-empathetic, distanced attitude and fail to pay attention to patients’ verbal and nonverbal communication (Halpern, 2014). This in direct contradiction to the ethics of nursing (American Nurses Association., 2015), including holistic nursing (Blaszko Helming et al., 2020) and can put patients’ safety at risk (Halpern, 2014).

### Mindfulness with Compassion Training

One way to help emergency department nurses constructively handle their stressful working environment might be to improve self-reflection and self-care via training in mindfulness, which includes compassion training. Mindfulness can be defined as paying attention to present-moment thoughts with an open and curious mind, experiencing, noting, and reflecting on feelings and sensations while accepting and not trying to change them (Baer, 2003; Kabat-Zinn, 2013). A typical mindfulness course includes approximately ten to twenty participants who attend a weekly two- to three-hour session for eight weeks. The course usually focuses on developing present-centered awareness by attending to sensory perceptions and bodily sensations such as breathing. A single, daylong silent retreat focused on practice is typically held after the sixth week, and the day includes a silent lunch (Santorelli et al., 2017). By improving nurses’ well-being, mindfulness may have the potential to facilitate holistic nursing care and help nurses provide empathetic, patient-centered care over the long term (White, 2014). Research in health care shows that mindfulness may increase compassion and reduce burnout in nurses (Blomberg et al., 2016; Guillaumie et al., 2017; Singer & Klimecki, 2014). Moreover, a questionnaire study of 50 emergency department nurses found that mindfulness was linked to lower symptoms of depression, anxiety, and burnout (Westphal et al., 2015).

### Previous Mindfulness Interventions Using Smartphone Apps

Mindfulness training, not least via smartphone, has grown in popularity in the last decade. However, at the time we designed and tested our intervention, only a few studies had investigated mindfulness training using these common applications (apps) in health care settings (dos Santos et al., 2016; Gauthier et al., 2015).

### The Intervention

**Figure 1.**
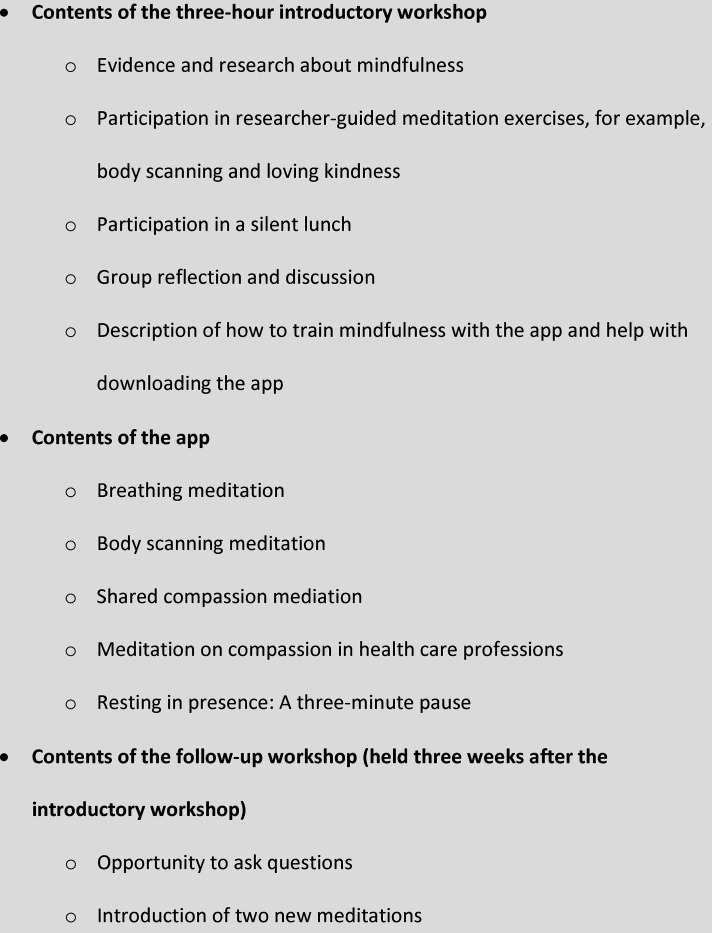
The mindfulness training intervention.

To investigate whether mindfulness training could increase compassion and empathy and lower stress in emergency department nurses, researchers from a medical university in Europe developed a mindfulness training intervention (Figure 1). It was based on the 8-week mindfulness-based stress reduction (MBSR) program developed by Jon Kabat-Zinn (Kabat-Zinn, 2013). The intervention was delivered via workshops and a series of meditations entitled “Mindfulness, empathy, and compassion for health care personnel” (Karolinska Institutet and MindApps, 2016). These meditations were available on the smartphone app “Mindfulness.” A research team that included one of the authors tested the intervention in an emergency department in a major metropolitan area between December 2016 and May 2017; 51 of the approximately 200 nurses and assistant nurses who worked in the department participated.

In brief, the nurses attended a three-hour workshop that focused on compassion, empathy, and mindfulness and included a silent lunch. The aim of the workshop was to provide an evidence-based introduction to and brief training in mindfulness. Participants had the opportunity to test body scanning and a loving kindness meditation. Body scanning involves systematically focusing on one body part at a time to increase your awareness of and contact with your body and yourself (Kabat-Zinn, 2013). Loving kindness meditation is a way to cultivate non-judgmental and compassionate loving kindness toward oneself and others (Boellinghaus et al., 2014; Salzberg, 1995). In this form of meditation, the participant focuses (for example) on experiences of love and care from people close to them and then gradually extends these feelings to others, including people they do not know. At the workshop, the researchers explained that the participants were to use one or more of the meditations in the app to practice mindfulness daily for eight weeks. The researchers then guided participants in using either the App Store or Google Play to download the app. At a follow-up workshop three weeks later, participants had the opportunity to ask questions about the intervention and its components, reflect on their experience so far, and try out two additional meditations.

### Aims

In the current study, we aimed to explore emergency care nurses and assistant nurses’ experiences of participating in the project to increase empathy and compassion through mindfulness training delivered via workshops and a smartphone app. We also explored how the nurses felt about the practical and technical aspects of the intervention.

## Methods

### Research Design, Setting, and Authors

This was a qualitative individual interview study of nurses working at an emergency department in a hospital in a major European city. The researchers used the phenomenological research method of systematic text condensation as described by Malterud (2012) to explore participants’ lived experiences.

The authors of the study are all women. The first author is an RN trained in qualitative interviewing and was a master's student at the time of the study. The second author is an RN with a PhD in medical science who participated in designing and conducting the mindfulness meditation intervention. The third author is an RN with a PhD in medical science and experience in qualitative methods.

### Participants

The 51 nurses and assistant nurses who took part in the intervention were invited by email to participate in the interviews. After three email reminders, five nurses and three assistant nurses (a total of eight individuals) who had taken part in the mindfulness intervention agreed to participate and were interviewed. Hereafter, all are referred to as “nurses.” Their ages ranged from 30 to 63 years, and they had worked in health care between 3 and 35 years. Seven were women and one was a man. For the sake of privacy and confidentiality, throughout this article, individual participants are referred to as “they” or “them.”

### Data Collection Methods

Participants in the intervention group were invited by email to take part in an individual interview. Interviews with those who agreed to participate took place 12 to 15 weeks after the start of intervention. The first author held the interviews in a quiet hospital break room near the emergency department where the participants worked. The interviews started with a general question or statement, such as “Please tell me what it was like to participate in the workshop and also try the app with exercises.” A semi-structured interview guide developed by the research group was used to ask supplementary questions as needed, for example, “What did you think of body scanning?” and “Is there anything else you want to tell me?” The interview also included questions about the practical and technical aspects of the intervention, for example, “What did you think of the silent lunch?” and “Did the app work for you (purely technically)?”

Before each interview, the interviewer reminded the participant of the purpose of the study and the voluntary nature of their participation. Participants’ identities were replaced with numbers at the time of the interview. The interviews lasted between 20 and 50 min, were audio recorded, and were transcribed verbatim. The authors transcribed two of the interviews, and a secretary transcribed the rest.

### Data Analysis

The first and second authors carried out the analysis in four steps as described by Malterud (2012). Malterud's method of systematic text condensation is based on Amedeo Giorgi's psychological phenomenological analysis. In phenomenological approaches, researchers attempt to suspend or “bracket” their presuppositions and look at the phenomenon of interest from the perspective of the people who experience it, thus allowing the essence of the phenomenon to emerge. The first step in systematic text condensation is to obtain an overall impression and develop preliminary themes (Malterud, 2012). During this step, the first and second authors read all the interview transcripts with an awareness of their prior understanding and impulses to systematize. They identified preliminary themes relevant to the study aim. In the second step of Malterud's process of systematic text condensation, researchers use the preliminary themes as a guide to identify and sort meaning units (parts of the text relevant to the research question) (Malterud, 2012). In this step, the first and second authors re-read the transcripts line by line. They highlighted meaning units, typically sentences, relevant to the study aims and discarded the parts of the interview that were not relevant. They lifted the meaning units out of their original context. Using the preliminary themes as a guide, they marked each meaning unit with a code that allowed them to gather the units into related groups. The third step in Malterud's process of systematic text condensation is to condense the meaning of the units in each code group (Malterud, 2012). To carry out this step, the first and second authors created a matrix that showed all the meaning units, by participant, that were relevant to each code. They then abstracted the meaning units under each code into an artificial quotation called a condensate. This artificial quotation summarized the essence of what participants said about the topic. The fourth step in systematic text condensation moves from condensation back to descriptions and concepts. Malterud refers to this as “putting the pieces together again” (Malterud, 2012). In this step, the first and second authors grouped the condensates under the themes and gave each theme a title that expressed the content of the theme. They then validated the findings by re-reading the transcripts to check the findings against the content of the transcripts. As part of this step, the researchers identified quotations from participants that illustrated each theme and condensate.

In keeping with Malterud's method (2012), the work was carried out flexibly, meaning that the researchers read and worked through the material several times and that the matrix was continuously updated. To improve trustworthiness, the authors regularly discussed the themes and their contents with each other. The third author joined the group near the end of analytical process. She had not participated in the intervention or in the interviews, so she was able to provide a new perspective during the final phase of analysis.

### Ethical Considerations

Ethical approval for the study was obtained from the Regional Ethical Review Board in Stockholm (Dnr 2015/5: 9; 2017 / 456-32). All participants were informed verbally about the purpose of the study and about their right to withdraw from the study at any time without explanation or consequences. They were also informed that they would remain anonymous in the presentation of the results. Consent was provided verbally.

## Findings

Three themes summarize the findings (Table 1). Two themes expressed nurses’ experiences of participating in the project to use mindfulness to increase empathy and compassion and decrease stress: *becoming aware* and *changing through mindfulness*. The third theme, *gaining the tools for mindfulness through workshops and the mobile app*, expressed nurses’ experiences of the practical and technical aspects of the intervention.

**Table 1.** Themes and Their Condensates (Subthemes)

Theme 1: Becoming aware	Theme 2: Changing through mindfulness	Theme 3: Gaining the tools for mindfulness through workshops and the mobile app
Gaining insight into one's own situation	Gaining acceptance of one's own situation	Learning about the scientific basis for the method
Discovering the need to reflect on and discuss behaviors	Becoming more able to center and focus	Gaining insight through eating in silence and being together
	Experiencing increased energy	Using the mobile app
	Experiencing the benefits of improved attitude and behavior	Making time and space for mediation
		Establishing and maintaining a new meditation routine

### Theme: Becoming Aware

Two subthemes reflected the nurses’ experiences of awareness that grew through mindfulness and meditation.

### Theme: Becoming Aware

#### Subtheme: Gaining Insight into One's Own Situation

Meditation led to insight, clearing the way for the nurses to become aware of aspects of their own situation that they previously had not recognized. For instance, those who meditated noticed the constant chatter of their thoughts: “Depending on how stressed you are […] [your] thoughts spiral out of control” (Participant 2). They became aware of how difficult it could be to meet their own needs during a busy rotation. Examples included the need for a calm meal break or even a bathroom break, and the need to focus on one thing at a time. “And sometimes when it was really awful, and there's too much coming from all directions, this three-minute exercise could actually be like really good, so focus now, you can only do one thing at a time” (Participant 7). They also described how they started to notice their attitudes toward and treatment of patients and colleagues. For example, one participant described her discomfort and deep sadness over discovering that she no longer felt empathy with patients but rather great irritation.

### Theme: Becoming Aware

#### Subtheme: Discovering the Need to Reflect on and Discuss Behaviors

Increased awareness led participants to reflect on their own attitudes and behaviors and those of colleagues. They were interested in and felt the need to discuss their reflections with their fellow nurses. For example, they thought and talked about how their behavior changed when they were stressed and how seeing colleagues display a less-than-adequate attitude, demeanor, or behavior toward patients could create stress. One participant said, “You also need to reflect on your own everyday actions and think about these things, especially about meeting other people. How do we treat each other and how do we treat our patients?” (Participant 2).

Some topics were difficult to assimilate and to discuss. For example, the nurse who had the insight that they felt irritation rather than empathy toward patients felt ashamed and at first unwilling to tell the others about these feelings. However, the workshop included dialogue about feelings such as these. After hearing that others had had similar experiences, the nurse felt able to discuss their own experience of irritation and decreased empathy. After reflection, the nurse linked this experience with stress and high workload, and their feeling of shame diminished.

Another topic of reflection and discussion was the insight that the whole working day could go by without any rest, which could leave the participants exhausted and overwhelmed. At breaks, for example, the nurses felt that they were expected to socialize with others even if they would have preferred not to. One said, “For example, at the emergency department, with such a large personnel group, we had, there are also so many in the personnel room that also when we sit there, if you’re not holding your telephone, it feels like a must, you have to talk …” (Participant 4).

### Theme: Changing Through Mindfulness

Not all participants managed to follow the intervention as intended. However, those who succeeded in finding a way to meditate during the intervention reported positive experiences of change. Four subthemes were found.

### Theme: Changing Through Mindfulness

#### Subtheme: Gaining Acceptance of One's Own Situation

The participants who meditated regularly described how the meditation toned down their thoughts and cleared their minds, which in turn meant that their stress and anxiety decreased. They also had a feeling of being mentally refreshed. These were helpful experiences, particularly those days when their work was especially heavy. The effect could occur as soon as they started the exercise. One said, “… it's very nice to meditate. Afterward you like clean up somehow. You start blank again and cope and keep up and so much more after, when you’ve mediated” (Participant 1).

Several developed a more accepting attitude toward themselves and their ability to handle their workload, which had a positive effect on their mood and reduced their feelings of stress. One participant said, “I feel that I’m much calmer. I have more acceptance that there's a lot of pressure, and I feel that I can handle it. It's very nice” (Participant 8).

With the help of mediation, it became easier to leave thoughts about work behind and let go of the workday. It was easier to adjust to leisure time, something nurses said that even their families and friends noticed.

### Theme: Changing Through Mindfulness

#### Subtheme: Becoming More Able to Center and Focus

Several participants described the constant input, events, and human interactions that take place at an emergency department as stressful at times. This hectic environment could also make it difficult for them to prioritize what was most important. The participants who regularly meditated during the study explained that they became more able to center themselves, be present, and focus on doing one thing at a time: “I have like a little break. I collect myself before treating patients. Yeah, then you can say that I meditate at work. Just a little while, I try to center myself and be here and now, collect everything” (Participant 1). Participants described greater awareness of where they focused their energy and greater awareness of the moment, both of which reduced the feeling of stress.

### Theme: Changing Through Mindfulness

#### Subtheme: Experiencing Increased Energy

Several participants were surprised that a short period of inward focus could change their energy level and help them manage more. They described their working days as full of many impressions, decisions they had to make quickly, and often a feeling of tiredness and lack of energy during their free time. One participant explained that to start with, they had a constant feeling of not doing a sufficiently good job: demands were consistently higher than their ability to meet them, and this took a lot of energy. However, after the intervention, the participant could handle work more easily, felt more satisfied, and had the energy to take part in social activity during free time. “I can go home now and do what I want and still have energy for it” (Participant 8).

### Theme: Changing Through Mindfulness

#### Subtheme: Experiencing the Benefits of Improved Attitude and Behavior

The nurses stated that the harsh climate common at the emergency department could lead to stress, a poor attitude and poor behavior toward others, and even complaints from patients. Regardless of whether a nurse behaved poorly toward someone or witnessed such behavior, it could induce stress and job dissatisfaction. “It isn't okay to get written complaints because of a poor attitude and behavior,” said one participant (Participant 2).

The mediations led to reflection and behavioral changes that were reinforced after participants noticed that the changes had positive effects. Examples of changed behavior included being more present, looking patients and colleagues in the eye, saying hello to coworkers in the corridors, and adjusting demeanor toward patients:

If I go in, switch off [the call button], and maybe stand at the edge of the bed, then I also notice there's a huge difference. And I also notice that it's not that it takes longer but only that you stand a little closer and don't stand in the doorway. The fact that I allow myself to take that time and go in, stand next to the bed and have that approach to the patients, I feel has given me a lot just that I don't stand in the doorway. (Participant 8)

As a result of the mindfulness intervention, it was easier for the nurses to focus on the patients they were with and not on what they needed to do later. Participants described how being present in the moment increased patient satisfaction, as well as leading to less stress for the patients and the nurses and a more satisfactory work situation. One nurse said, “This mindfulness, it's about, it's obviously [helpful] for the patients, but it's also about us—that we should feel that we’re satisfied. Do I feel that I’ve done what I could?” (Participant 6).

### Theme: Gaining the Tools for Mindfulness Through Workshops and the Mobile App

The third theme described how the nurses felt about the practical and technical aspects of the intervention. Five subthemes were found.

### Theme: Gaining the Tools for Mindfulness Through Workshops and the Mobile App

#### Subtheme: Learning about the Scientific Basis for the Method

The thorough start of the intervention, a three-hour workshop, was important. During the workshop, two of the researchers provided information about the scientific basis of the mindfulness method, which helped give the participants the feeling of security they needed to participate. “Yes, I thought the workshop was good. There were consistent background facts about why they wanted to start this and what was found in previous research” (Participant 7). One participant explained that she would never have dared to try mindfulness without the study: “For me, it was a little more concrete that this isn't nonsense, this isn't something invented that someone has [just] come up with, but that there's actually a fairly scientific basis for this to actually work for many [people]” (Participant 8).

### Theme: Gaining the Tools for Mindfulness Through Workshops and the Mobile App

#### Subtheme: Gaining Insight Through Eating in Silence and Being Together

Participants highlighted the importance of two aspects of the workshop to gaining insight and awareness. The first was the silent lunch. According to participants, this interesting experience was difficult in the beginning but quite comfortable after a while. They described insights they had about themselves during the lunch.

Being silent with other people wasn't something uncomfortable or strange in any way but was … yeah … there was actually a kind of fellowship in that we all did the same thing and we did it together. We ate together. That's fellowship in and of itself even if we didn't talk with each other … instead it was more the revelation of how hyper I was … the tempo I have … (Participant 5)

The second aspect of the workshop that participants highlighted was the importance of taking part in the intervention together. This enabled them to discuss their shared experiences. Reflecting on nursing care in this way was important, exciting, and helped participants see things from other points of view. “I thought the workshop itself was quite good, because in part it was good to get some other perspectives” (Participant 6).

### Theme: Gaining the Tools for Mindfulness Through Workshops and the Mobile App

#### Subtheme: Using the Mobile App

Not all participants used the meditation app as instructed. Instead of navigating to the free Swedish app on their cell phone, one nurse accidentally navigated to a payment screen in English, and this led the nurse to give up. Another explained that they did not use the app because they did not have the energy to learn how it worked. Moreover, they had another meditation habit that they preferred. A third found their own way to meditate that did not involve the app. However, the others felt that the app was easy to download and use.

Body scanning was the most-used exercise. Participants explained that this was because of its effects, such as better sleep, less stress, a feeling of getting to know your body, and an ability to relax more easily. “I use body scanning quite a lot before going to sleep. I was a little stressed. Then I found that I got a little calmer the nights when I did it [body scanning]” (Participant 2). Participants also said that the body scanning exercise was of a reasonable length and easy to grasp.

The voice in the guided meditation was important and something several participants mentioned spontaneously. A calm, pleasant, and friendly voice made it easier. One explained, “But a lot also depends on their voices, that they could get you to relax and you could like go [inward] into yourself” (Participant 6).

### Theme: Gaining the Tools for Mindfulness Through Workshops and the Mobile App

#### Subtheme: Making Time and Space for Meditation

It was hard for most participants to find space and to take the time to meditate during working hours. “I probably would have benefited if I interrupted my day, paused for a moment during the day at work, but I never gave myself the time to do it” (Participant 5). Only one participant managed to meditate regularly at work. Participants said that the need to remain reachable at all times, which was part of their job, was an obstacle to meditating at work. Another obstacle was the lack of a place where they could feel confident that they would be alone.

Most participants meditated at home after or just before work, in bed or on the sofa. Some practiced on the bus on the way home from work. Participants said that to be sure they would actually do the exercise, it was important to decide ahead of time when they would do it. They explained that certain thoughts could help motivate them to meditate. These included the thoughts that meditation could help them manage to keep working in the stressful emergency department environment and could facilitate continued good health. “You just need to give yourself time [to meditate]. And you’re worth that time […] you’re worth feeling well” (Participant 8).

Not everyone used the app, and not everyone used the mindfulness activities daily. Use of the app was highest at the beginning of the study and after the follow-up meeting. Several of the participants said that in retrospect, they wished that they had practiced more regularly.

### Theme: Gaining the Tools for Mindfulness Through Workshops and the Mobile App

#### Subtheme: Establishing and Maintaining a New Meditation Routine

Some participants explained that they meditated regularly before the study, but for the others, meditation was completely new. Getting into the habit of doing the meditations could be difficult. One nurse explained, “I tried the app in the beginning a bit, tried to be a bit regular, but it's failed a little now at the end, now I haven't done it in a long time” (Participant 3).

Participants described problems that made it challenging to establish the new habit. They could feel irritated that there was a lot of talking in some of the meditations, which made it harder for them to relax. They could find it difficult and unfamiliar to sit still and meditate. However, their ability to do so increased as they continued to practice regularly: “I also noticed from the beginning that after maybe two minutes I feel a little annoyed, then I stop, and next time I do it again, then it's a little more than two minutes, but finally I can do it five to six” (Participant 4). Perseverance and finding a personal favorite meditation helped participants succeed in establishing a routine.

## Discussion

In this qualitative interview study of emergency care nurses’ experiences of a mindfulness training intervention, we found that participants could become more aware of their own situation and behaviors. Not everyone was able to meditate regularly, but those who did could experience change through mindfulness. They described gaining acceptance, increasing their ability to center and focus, gaining more energy, and improving their attitude and behavior toward patients and colleagues. Some of the changes, especially improvements in attitude and behavior, led to benefits for both patients and nurses. The information on the scientific basis of mindfulness, provided in the workshop, gave the nurses the level of trust they needed to undertake mindfulness training. Reasons for not adhering to the intervention included difficulties with the app and with finding time and space to meditate during working hours.

### Becoming Aware

As a nurse, caring for one's own needs is a prerequisite for providing good and holistic care (Blaszko Helming et al., 2020; Petleski, 2013; Rathert et al., 2016). However, to care for their own needs, nurses first need to become aware of them. In this study, the mindfulness intervention helped emergency department nurses achieve such awareness. During the intervention, nurses became aware of several different sources of stress, such as the constant chatter of their thoughts and the difficulty of meeting their own needs during a busy rotation. Researchers from Australia who investigated emergency department nurses’ experiences of a mindfulness-based self-care and resiliency intervention found that nurses gained perspective and insight into their situation (Slatyer et al., 2018b).

Another important source of stress in the current study was observing fellow nurses encounter patients or other nurses in a way that was not empathetic, which negatively influenced the caring environment. The caring environment is one of the many components that come together to enable nurses to provide holistic care (Blaszko Helming et al., 2020). When a caregiver's intention to provide quality care conflicts with the system or culture, the caregiver can experience moral distress and ethical dilemmas (AHNA, 2021; Rushton, 2006). Indeed, during the intervention, nurses could become aware that they had lost empathy with patients, a discovery that prompted feelings of surprise, sadness, and discomfort.

Among the most meaningful parts of the mindfulness intervention was discovering the importance of reflection, a competency essential to holistic nursing (Blaszko Helming et al., 2020; Frisch, 2001). Through individual and group reflection, nurses were able to recognize and therefore process difficult feelings and thoughts about their work. A systematic review of qualitative studies of mindfulness interventions for health care workers also found that the experience of meeting in a group and discovering that they were not alone in their experiences of difficulties was a crucial benefit of the interventions (Morgan et al., 2015). A later study of emergency department nurses in Australia (Slatyer et al., 2018b) underscores these findings. In that study, nurses liked the group format of the intervention because it allowed them to reflect together, learn from each other, and feel less isolated or alone (e.g. in experiencing stress). Research shows that negative feelings and behaviors in the working environment can be changed by increasing people's awareness of them through reflection and discussion (Singer & Klimecki, 2014). Reflection also helped the nurses recognize and process thoughts and feelings about the ways they interacted with patients. This is consistent with prior research showing that mindfulness helps emergency department nurses broaden their perspective on interpersonal meetings with patients, which is a prerequisite for providing empathetic (Salvarani et al., 2019) and holistic care (Blaszko Helming et al., 2020).

### Changing Through Mindfulness

Our finding that nurses who used the mindfulness app regularly could experience change is consistent with the findings of a 2019 meta-analysis of randomized controlled trials that found that mindfulness training could reduce distress and improve well-being in health care professionals (Spinelli et al., 2019). Subsequent research has led to similar findings (Sarazine et al., 2021).

#### Gaining Acceptance of One's Own Situation

Participants described how awareness of their own reactions and stress levels improved their ability to cope with their workday, a finding consistent with the core value that self-care is crucial to providing good holistic care. This is in keeping with the findings of a systematic review of brief mindfulness practices for health care providers, which found that these practices increased mindfulness and reduced stress and anxiety (Gilmartin et al., 2017). A previous quantitative study also found that in nurses working in intensive care, mindfulness was associated with lower levels of stress and could be a resource for coping with stress (Lu et al., 2019). This may be because after meditation, nurses feel that they can more clearly analyze complex situations and regulate their emotions in stressful contexts (Guillaumie et al., 2017; Morgan et al., 2015). Similarly, after using a mindfulness meditation app, medical students reported reduced stress (Yang et al., 2018). Other studies have reported increased self-compassion in nurses following mindfulness meditation interventions (Gracia Gozalo et al., 2019; Slatyer et al., 2018a), one of which incorporated support via smartphone groups (WhatsApp) (Gracia Gozalo et al., 2019).

#### Becoming More Able to Center and Focus

In our study, nurses who regularly mediated described being more aware of the moment, increased ability to focus their energy, and improved ability to focus on doing one thing at a time. In previous studies, emergency department nurses described feelings of inner calm and a better ability to focus and think clearly following a mindfulness meditation intervention (Slatyer et al., 2018b), and nurses who mediated regularly found that they had more focus (Smith, 2014). A meta-analysis found that calming and slowing the mind and increased ability to choose what to focus on was a common theme in qualitative studies of health care professionals’ experiences of mindfulness meditation (Morgan et al., 2015). A qualitative study of people in an urban setting similarly found that using a mindfulness app could lead to rewarding feelings of “being in the moment” and the ability to look at things from a different perspective (Laurie & Blandford, 2016). There is some support for this finding from quantitative studies, as well. A meta-analysis of randomized controlled trials found evidence that mindfulness smartphone apps can teach mindful awareness and acceptance of the present moment (Linardon, 2020).

#### Experiencing Increased Energy

The nurses in the current study reported that a short period of inward focus could change their energy level and help them handle more, as well as give them more energy to take part in social activities during their free time. These findings are in keeping with those of a previous mindfulness intervention that found that nurses who mediated regularly had more energy (Smith, 2014). Another study has found that nurses report that meditation can increase their feelings of enthusiasm (Guillaumie et al., 2017).

#### Experiencing the Benefits of Improved Attitude and Behavior

We found that participants who meditated regularly experienced a positive change in their attitude toward colleagues and patients and greater satisfaction with work. Similar findings were reported in a mixed-methods systematic review of the effect of mindfulness on nurses (Guillaumie et al., 2017). The qualitative studies in the review found that after the intervention, nurses described improved communication with colleagues and patients and greater sensitivity to patients’ experiences. The authors of the systematic review called for more research to explore the impact of mindfulness on nurses’ work performance. At least one subsequent study found that a mindfulness-based intervention increased not only emergency department nurses’ mindfulness, but also patient satisfaction (Saban et al., 2021). Those findings, like ours, are consistent with previous results showing that mindfulness meditation has biological and psychological effects that positively impact attitudes and behaviors (Conklin et al., 2019). In the current study, nurses described behavioral changes that were reinforced after they noticed that the changes had positive effects: being more present, looking patients and colleagues in the eye, saying hello to coworkers in the corridors, and adjusting demeanor toward patients.

Participants in our study noted that even during brief moments, such as drawing blood, there was always space to show presence—for example, by listening carefully to the patient. This theme or concept has been found in other qualitative studies of mindfulness interventions for health care professionals (Morgan et al., 2015). Listening closely is one way to display empathy in a clinical setting (Halpern, 2014). This could be interpreted as showing increased compassion. Other studies have reported more compassion (Guillaumie et al., 2017), self-reported compassion (Morgan et al., 2015; Sanko et al., 2016), and empathy (Smith, 2014) following mindfulness meditation (Pagnini & Philips, 2015). A quantitative study of mindfulness meditation for pediatric nurses similarly found increases in “acting with awareness” (being aware of actions in the present moment), which was in turn associated with compassion satisfaction (good feelings arising from helping other people) (Morrison et al., 2017). Another study of mindfulness meditation in hospital nurses also showed that the intervention increased compassion satisfaction (Slatyer et al., 2018a). However, a review of 24 studies of mindfulness meditation in nursing care found that it was difficult to draw conclusions about the effectiveness of mindfulness given the current state of inquiry (Blomberg et al., 2016).

### Gaining the Tools for Mindfulness Through Workshops and the Mobile App

#### Learning About the Scientific Basis for the Method

We found that nurses wanted reassurance that mindfulness training had a scientific basis and was not “nonsense.” Other researchers have also found that participants can need to overcome negative perceptions of mindfulness (Laurie & Blandford, 2016), such as the concern that it is not evidence-based (Clarke & Draper, 2019).

#### Using the Mobile App

Not all participants used the meditation app as instructed. Choosing not to adhere to or complete mindfulness meditation interventions is common (Cavanagh et al., 2014; Gál et al., 2021; Parsons et al., 2017; Shapiro et al., 2005). For example, in one large study of a mindfulness intervention using a mobile app, only 24% of participants completed the intervention (Mak et al., 2018). It is possible that addressing the common difficulty of finding time and space to meditate, discussed below, could improve adherence.

Body scanning was the most popular exercise in the current study because it had calming effects, was of reasonable length, and was easy to grasp. In contrast, in another study, participants highly valued meditations that focused on breathing (Laurie & Blandford, 2016). It difficult to draw conclusions from just two studies, but in future interventions, it may be appropriate to provide a variety of exercises so that participants can choose the kind that suits them best.

Studies of meditation apps are making it increasingly clear that meditators have preferences regarding narrators’ voices and the amount of talking in guided meditations (Clarke & Draper, 2019; Laurie & Blandford, 2016). According to the nurses in our study, a calm, pleasant, and friendly voice made it easier to use the guided meditations and to relax. This echoes the findings of other qualitative studies of meditation apps, which also reported that the speaker's voice was important to participants (Clarke & Draper, 2019; Laurie & Blandford, 2016). Like participants in a previous study (Clarke & Draper, 2019), the nurses in the current study could also feel that lots of talking in a meditation made it hard to relax.

#### Making Time and Space for Meditation

Finding the time and space to meditate is a main barrier to adhering to meditation interventions (Laurie & Blandford, 2016; Shapiro et al., 2005; Wyatt et al., 2014). Our study and others have found that is also true for health care professionals, both at home and at the workplace (Banerjee et al., 2017; Morgan et al., 2015; Slatyer et al., 2018b). Scheduling a daily workplace meditation session as part of interventions might be a way to counteract this difficulty. A previous pilot study in a pediatric intensive care unit found that a five-minute mindfulness training session at the start of the work shift made it feasible to meditate at work (Gauthier et al., 2015).

Participants in the current study who managed to meditate most often did so on the way to or from work, just before work, or at home after work. This finding is consistent with the findings of a previous qualitative study of a meditation app (Laurie & Blandford, 2016). That study found that participants usually meditated at home or on public transportation.

#### Establishing and Maintaining a New Meditation Routine

Finally, getting into the habit of meditating regularly could be challenging, a finding that may stem, at least in part, from the difficulty of finding the time and space to meditate. A brief, in-person regular guided workplace meditation of the kind described in the pilot study of pediatric intensive care nurses might be a way to support the establishment of such a routine (Gauthier et al., 2015).

### Methodological Considerations

In this study, we chose to conduct individual interviews with participants to gain insight into their experiences. Another option would have been to conduct focus groups discussions. However, the group dynamics in focus group discussions can make it more difficult for participants to express divergent views (Malterud, 2012). In this study, we were particularly interested in identifying not only similarities but also differences in experiences.

Several aspects of the study affected its trustworthiness. The first author, who conducted the interviews, was not involved in the intervention and did not work at the hospital or know any of the participants in the study. The interviewer also used an interview guide with questions and follow-up questions that were open-ended to increase dependability. In the manuscript, representative quotations were used to illustrate the findings.

The time span between the intervention and the interview may have reduced participants’ ability to remember their experiences of the intervention. However, the delay may have also given them time to reflect on their experiences. The findings were not presented to participating nurses or other nurses to learn whether they recognize the findings; this affects creditability.

We strove for transparency in the presentation of the methods, for example, by describing each step in the analytical process. To increase confirmability, the first and second authors read the transcripts of each interview several times and compared transcripts with recordings. Throughout the analytical process, they wrote down their own reflections and thoughts. The first and second authors had prior understanding of nursing, mindfulness, and the intervention. The third author had prior knowledge of nursing but not of mindfulness or the intervention. Prior knowledge can aid in the interpretation of findings, but it can also have an influence on the analytical process (Polit & Beck, 2012). The researchers were aware of their prior understanding and consulted each other continually throughout the process.

One limitation of the study is the small sample size. Only eight of the 51 participants in the intervention chose to take part in the interviews, and we do not know if those who chose not to participate were different in some way from those who participated. However, the participants had worked as nurses for different lengths of time (3 to 30 years) and had a variety of jobs (licensed practical nurses, RNs, and managers), which may have strengthened transferability. Additionally, one participant was a man, and the rest were women, which made the group approximately representative of the sex distribution in health care in the country where the study was conducted (Swedish National Board of Health and Welfare, 2019).

### Implications for Research and Practice

Using mindfulness may support emergency nurses in providing holistic and compassionate care in a stressful environment. The study showed that nurses in emergency care are vulnerable to stress and may benefit from the self-reflection and self-compassion that mindfulness training can engender. Training mindfulness may be one way to increase nurses’ awareness of their thoughts, workloads, and stress levels. We found that nurses practicing a few minutes of daily mindfulness meditation could gain acceptance of their own situation, become more able to center and focus, experience increased energy, and experience the benefits of improved attitude and behavior.

The COVID-19 pandemic has put additional pressures on health care professionals, and emergency department nurses are particularly affected. Several researchers have suggested that mindfulness meditation delivered via smartphone apps, such as the one used in this study, could be one tool for such health care professionals (Alexopoulos et al., 2020; Reyes, 2020).

### Conclusions

This qualitative study suggests that practicing mindfulness may help shift the mindset of nurses and assistant nurses, helping them gain insight into their work situation. They may also realize that they feel the need to reflect on and discuss their professional behaviors. The study also showed that nurses who practiced mindfulness meditation could experience positive changes, including increased acceptance of their working situation, increased ability to center and focus, more energy, and attitudes and behaviors that were more positive. At the same time, emergency department nurses have difficulty finding the time and space to meditate, especially at work, and establishing a meditation routine.
